# Amide proton transfer-weighted habitat radiomics: a superior approach for preoperative prediction of lymphovascular space invasion in cervical cancer

**DOI:** 10.3389/fonc.2025.1599522

**Published:** 2025-07-10

**Authors:** Jie Li, Yatong Li, Lianze Du, Qinghai Yuan, Qinghe Han

**Affiliations:** Department of Radiology, The Second Hospital of Jilin University, Changchun, China

**Keywords:** habitat radiomics, amide proton transfer-weighted MRI, lymphovascular space invasion, cervical cancer, predictive modeling

## Abstract

**Background:**

Non-invasive preoperative prediction of lymphovascular space invasion (LVSI) in cervical cancer (CC) is clinically important for guiding surgical planning and adjuvant therapy, while avoiding the risks associated with invasive procedures. However, current studies using amide proton transfer-weighted (APTw) MRI for LVSI prediction typically analyze only the mean values from a limited number of intratumoral regions of interest (ROIs), which fails to fully capture tumor heterogeneity. This study investigates the added value of whole-tumor APTw habitat radiomics in predicting LVSI and its advantages over conventional analysis methods.

**Methods:**

This prospective study included consecutive adult patients with suspected CC who underwent APTw MRI between December 2022 and December 2024; a portion of the cohort has been reported previously. APTw values were extracted using two methods: (1) the conventional approach, calculating the mean signal from three ROIs on a representative slice; and (2) habitat radiomics, involving whole-tumor segmentation, k-means clustering to identify functional subregions, and radiomic feature extraction. Pathological assessment of LVSI from hysterectomy specimens served as the reference standard. Multivariable logistic regression identified variables associated with LVSI and developed diagnostic models. Model robustness was evaluated by 5-fold cross-validation, with AUC and DeLong’s test used for performance assessment.

**Results:**

Among 124 patients (74 LVSI−, 50 LVSI+), the APTw_h3 model achieved a higher AUC (0.796 [95% CI: 0.709–0.882]) for predicting LVSI positivity than the clinical-radiological model (AUC = 0.733, 95% CI: 0.638–0.817). The combined model integrating clinical, radiological, and APTw_h3 features achieved the highest AUC (0.903, 95% CI: 0.841–0.952), which was significantly higher than those of both the clinical-radiological and APTw_h3 models (both *P* < 0.001). Moreover, the addition of APTw_h3 to the clinical-radiological model improved sensitivity (88% vs. 82%) and specificity (83.8% vs. 64.9%) for determining LVSI positivity.

**Conclusion:**

Whole-tumor APTw habitat radiomics demonstrates superior performance over conventional mean-value APTw analysis for preoperative prediction of LVSI in CC. Notably, integrating habitat radiomic features with clinical and radiological parameters further improves predictive accuracy, demonstrating potential for enhanced individualized patient management.

## Introduction

Cervical cancer (CC) is a significant public health burden, ranking as the fourth most common malignancy in women globally, with rising incidence and mortality rates in China (1). In the context of precision medicine, early and accurate risk stratification is essential for optimizing treatment and improving outcomes (2). Among clinicopathological factors, lymphovascular space invasion (LVSI) is a critical intermediate-risk marker that independently predicts lymph node metastasis, distant recurrence, and overall survival (3), and directly informs the indication for adjuvant therapies such as chemoradiotherapy (4). Proper identification of LVSI status is therefore pivotal for determining the intensity of postoperative management, as LVSI-positive patients often require more aggressive adjuvant therapies, while LVSI-negative patients may avoid unnecessary treatment and related complications, ultimately influencing survival and quality of life (5).

Currently, the assessment of LVSI relies exclusively on postoperative histopathology—the current gold standard—which is invasive and limited to surgical specimens (6). This approach has several limitations: (1) it is not available in the pre-treatment setting, thereby precluding its use in initial treatment stratification (7); (2) it can delay subsequent therapy due to lengthy diagnostic processes (8); and (3) it cannot fully assess tumor heterogeneity (9). Although imaging techniques like MRI and PET offer complementary value in staging and planning (10), there is no validated imaging biomarker for reliably predicting LVSI before surgery. Development of such a non-invasive biomarker would greatly enhance individualized risk assessment and therapy planning (11).

Amide proton transfer-weighted (APTw) magnetic resonance imaging has recently gained attention as a novel molecular imaging approach (12). APTw imaging enables noninvasive, real-time quantification of amide proton exchange, indirectly reflecting tissue protein concentration and microenvironmental pH, two factors that are closely associated with tumor aggressiveness and LVSI (13). Some preliminary studies have suggested correlations between APTw-based parameters and LVSI status, raising its potential as an imaging biomarker (14). However, most studies have used analyses restricted to single manually segmented two-dimensional regions of interest (ROIs) on the largest tumor slice, extracting mean signal values or limited histogram features (15). This method cannot reflect the three-dimensional spatial heterogeneity inherent to tumors and may limit predictive performance—a limitation highlighted in our previous work on parametrial invasion (PMI) (16).

It is important to note that LVSI and PMI serve fundamentally different roles in CC management. While LVSI is critical for determining the need for adjuvant systemic therapy due to its strong association with metastatic risk, PMI mainly impacts surgical planning and eligibility for fertility-sparing procedures, and is less relevant for decisions about adjuvant therapy (16). Consequently, there is a need for dedicated, LVSI-specific predictive models, rather than extrapolations based on other risk factors.

To address these gaps, habitat imaging radiomics analysis has been proposed. This method applies unsupervised clustering to partition the entire tumor volume into multiple subregions with distinct APTw signal profiles. Advanced radiomic features, including histogram, texture, and spatial metrics, can then be extracted from each habitat, enabling a detailed assessment of tumor heterogeneity. Proof-of-concept studies have shown that habitat features from APTw imaging can help predict tumor aggressiveness (17).

Therefore, this study aims to develop and validate an APTw-based habitat radiomics model for the noninvasive, preoperative prediction of LVSI in CC, providing a potential imaging biomarker to enhance risk stratification and guide individualized treatment. While not intended to replace standard histopathological assessment, such imaging approaches could complement clinical workflows—for example, by assisting in high-risk patient identification, therapy planning, and recurrence risk stratification—thus supporting more personalized and effective clinical management.

## Methods

### Patients

**Figure 1:**
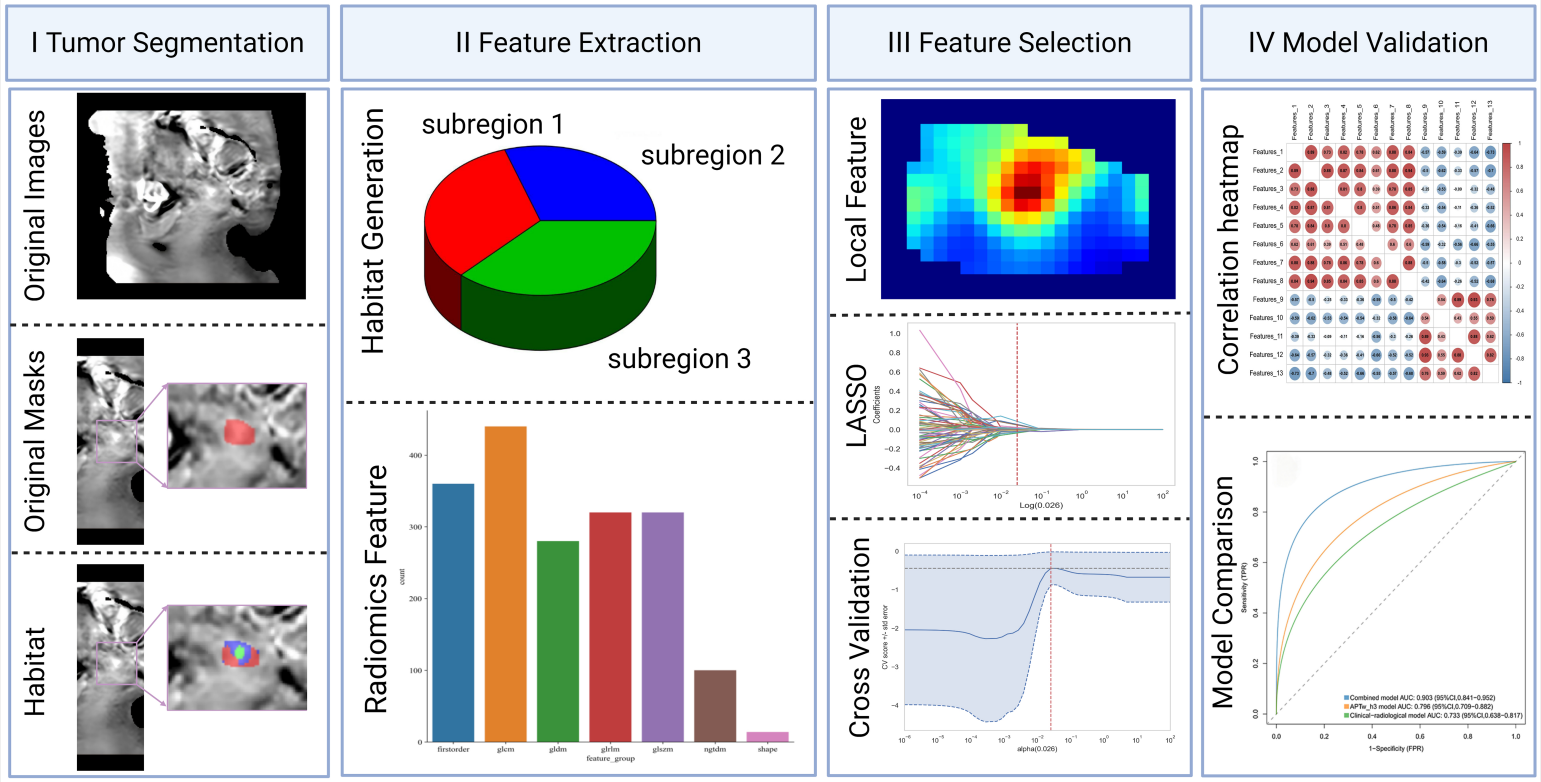
Schematic of the methodological framework.

**Figure 2:**
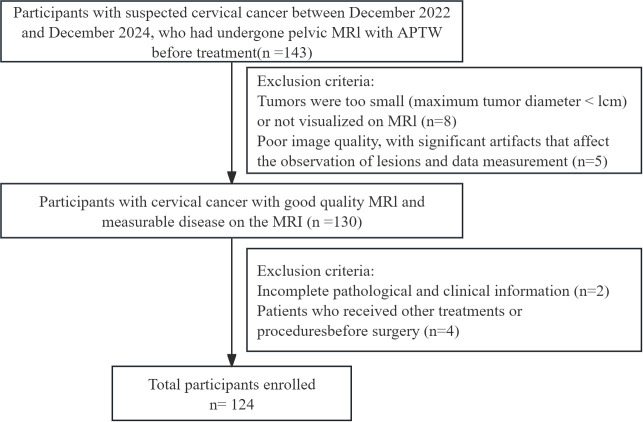
Flowchart of patient selection. APTw, amide proton transfer-weighted.

This is a prospective research study that adheres to the Declaration of Helsinki and was approved by the Ethics Committee of our institution, with approval number No. 2022-230. All participants provided written informed consent prior to imaging. From December 2022 to December 2024, adult participants presenting with clinical symptoms suggestive of CC underwent MRI and APTw imaging. This cohort partially overlaps with that used in our previous publication (10.21037/qims-24-412). The inclusion criteria were as follows: (1) surgically and pathologically confirmed CC and (2) preoperative pelvic MRI examination. The exclusion criteria were as follows: (1) tumor with a diameter less than 1 cm or not visible on MRI; (2) poor image quality with significant artefacts affecting lesion observation and data measurement; (3) incomplete pathological and clinical information; and (4) other preoperative treatments or interventions. The data and imaging records of 124 CC patients were included in this study. The following clinical data were collected from a review of the clinical case management system: age, body mass index, cancer antigen-125 (CA125) levels, menopausal status, and International Federation of Gynecology and Obstetrics (FIGO) stage. The methodological framework and the screening process are illustrated in **Figures 1** and **2**, respectively.

### Image acquisition

All patients were scanned with 3.0T MRI system (Ingenia 3.0 T CX; Philips Healthcare, Best, the Netherlands) equipped with an abdominal and pelvic phased-array coil. Patients were placed in the supine position with the bladder moderately filled. The scanning range extended from the umbilicus to the pubic symphysis. If the tumor was sufficiently large and involved a wide area, the scanning range was appropriately enlarged.

The routine MRI sequences included axial T1-weighted imaging, axial and sagittal T2-weighted imaging (T2WI), axial fat-suppressed T2WI, and dynamic contrast-enhanced MRI. Axial diffusion-weighted imaging (DWI) was additionally performed with b values of 50 and 800 seconds/mm². Apparent diffusion coefficient (ADC) images were automatically generated by the scanner using single-exponential fitting of the DWI data. Gadobutrol (trade name: Gadovist, produced by Hebei Hengrui) was used as the contrast agent and was administered at a dose of 0.1 mmol/kg body weight.

In APTw imaging, the APT value is obtained by calculating the percentage of magnetization transfer asymmetry (MTR_asym_) at a frequency offset of +3.5 ppm (16). The specific formula is as follows:


$$\text{APTw\%}={\text{MTR}}_{\text{asym}}\left[\text{Δω}=+3.5\text{ppm}\right]\left(\%\right)=\frac{{\text{S}}_{-\text{Δω}}-{\text{S}}_{\text{Δω}}}{{\text{S}}_{0}}\times 100\%$$

where S_-Δω_ and S_Δω_ represent the signals at frequency offsets of -Δω and Δω (Δω = +3.5 ppm), respectively; S_0_ represents the signal without radio frequency saturation. After the scan was completed, the APTw images were automatically generated on the console. The ADC map was generated on the basis of the DW images on the scanner console. Detailed information about the MRI and APTw imaging parameters is shown in **Table 1**.

**Table 1:** Magnetic resonance imaging instrument scanning parameters.

Parameter	TR (ms)	TE (ms)	FOV (cm)	Matrix	Layer thickness (mm)	Layer spacing (mm)	Scanning time (s)
Axial T1WI	500	13.13	30	332×289	4	1	47
Axial T2WI	2500	110	40	400×400	4	1	92
Sag T2WI	3500	110	30	300×300	4	1	77
DWI	4000	56.3	38	128×126	5	2	60
DCE-MRI	500	1.31	36	300×300	1.2	3	270
APTw	90	–	8	160×229	2	1	190

T1WI, T1 weighted imaging; T2WI, T2 weighted imaging; DWI, diffusion weighted imaging; DCE-MRI, dynamic contrast-enhanced magnetic resonance imaging; APTw, amide proton transfer-weighted; TR, repetition time; TE, echo time; FOV, field of view.

### Histologic analysis

According to the 2018 FIGO staging system, all participants underwent total hysterectomy, bilateral salpingo-oophorectomy, and surgical pathological staging (18, 19). The hysterectomy specimen was sliced along the vertical plane of the uterus; the depth of myometrial invasion was estimated during gross anatomical assessment and confirmed through microscopic evaluation using standard criteria. The uterus was sectioned at 3–4 mm intervals for further pathological analysis. Microscopic assessment was performed to confirm the tumor histological grade (low, 1 or 2; high, 3), parametrial invasion, and LVSI status. The presence of LVSI was determined via haematoxylin–eosin (H&E) staining; the LVSI status was recorded as positive when tumor emboli were observed in spaces lined by endothelium in the myometrium and outside the invasive front of the tumor; otherwise, the LVSI status was considered negative. All the samples were analyzed by a professional pathologist with 12 years of experience in urogenital pathology.

### Image processing

**Figure 3:**
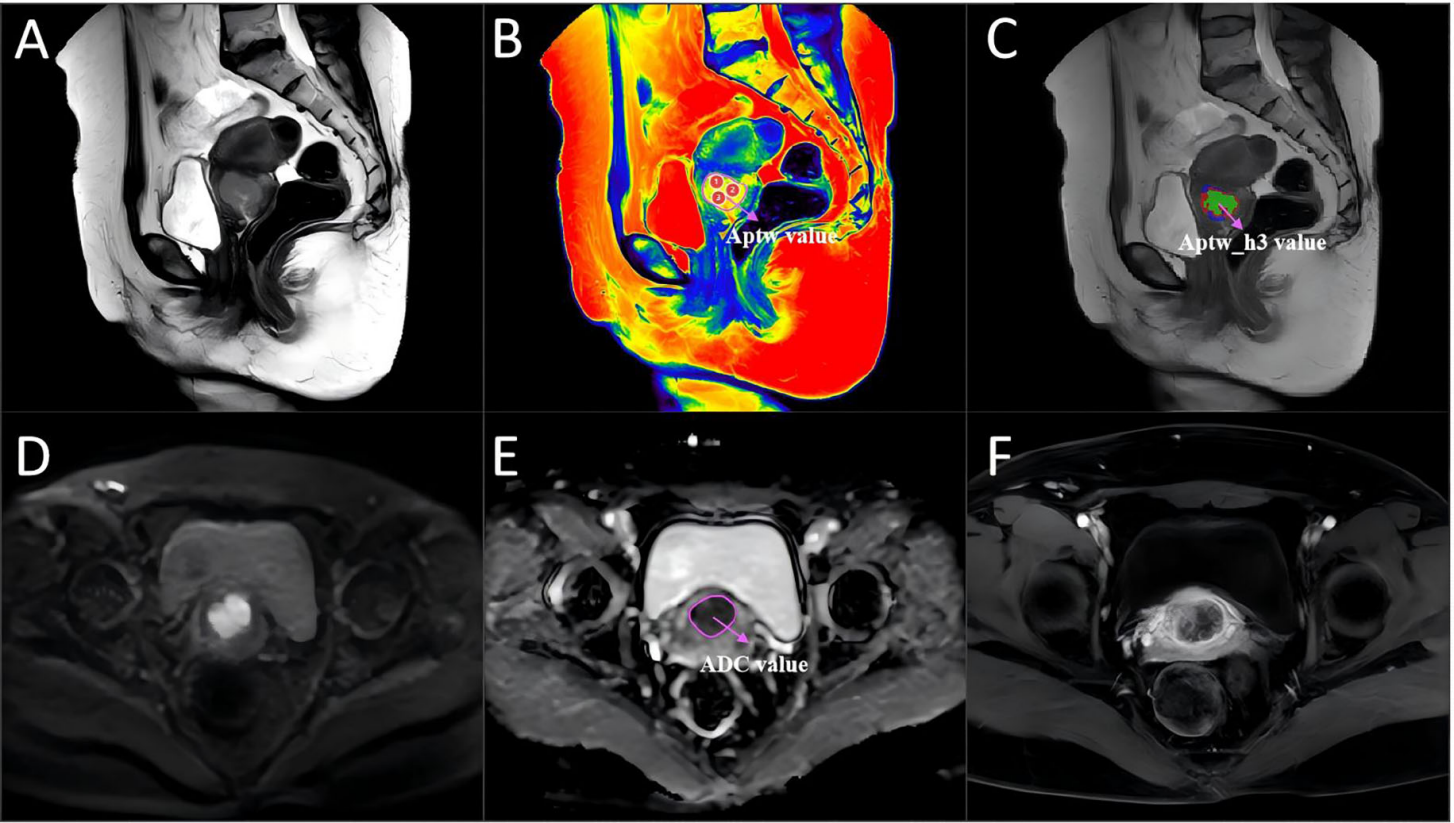
A 46-year-old woman with cervical cancer without LVSI, with a serum CA125 level of 16.8 U/mL and a largest tumor diameter of 31 mm. **(A)** Sagittal T2WI. **(B)** APTw image and T2W image fusion. **(C)** tumor region divided into three subregions via unsupervised clustering; red: subregion 1; blue: subregion 2; and green: subregion 3. **(D)** Diffusion-weighted image (b = 800 s/mm^2^). **(E)** ADC image. **(F)** DCE-MR image. The mean APTw and ADC values measured by the two radiologists were 2.31% and 1.74×10^−3^ mm^2^/sec, respectively. APTw, amide proton transfer-weighted; LVSI, lymphovascular space invasion.

**Figure 4:**
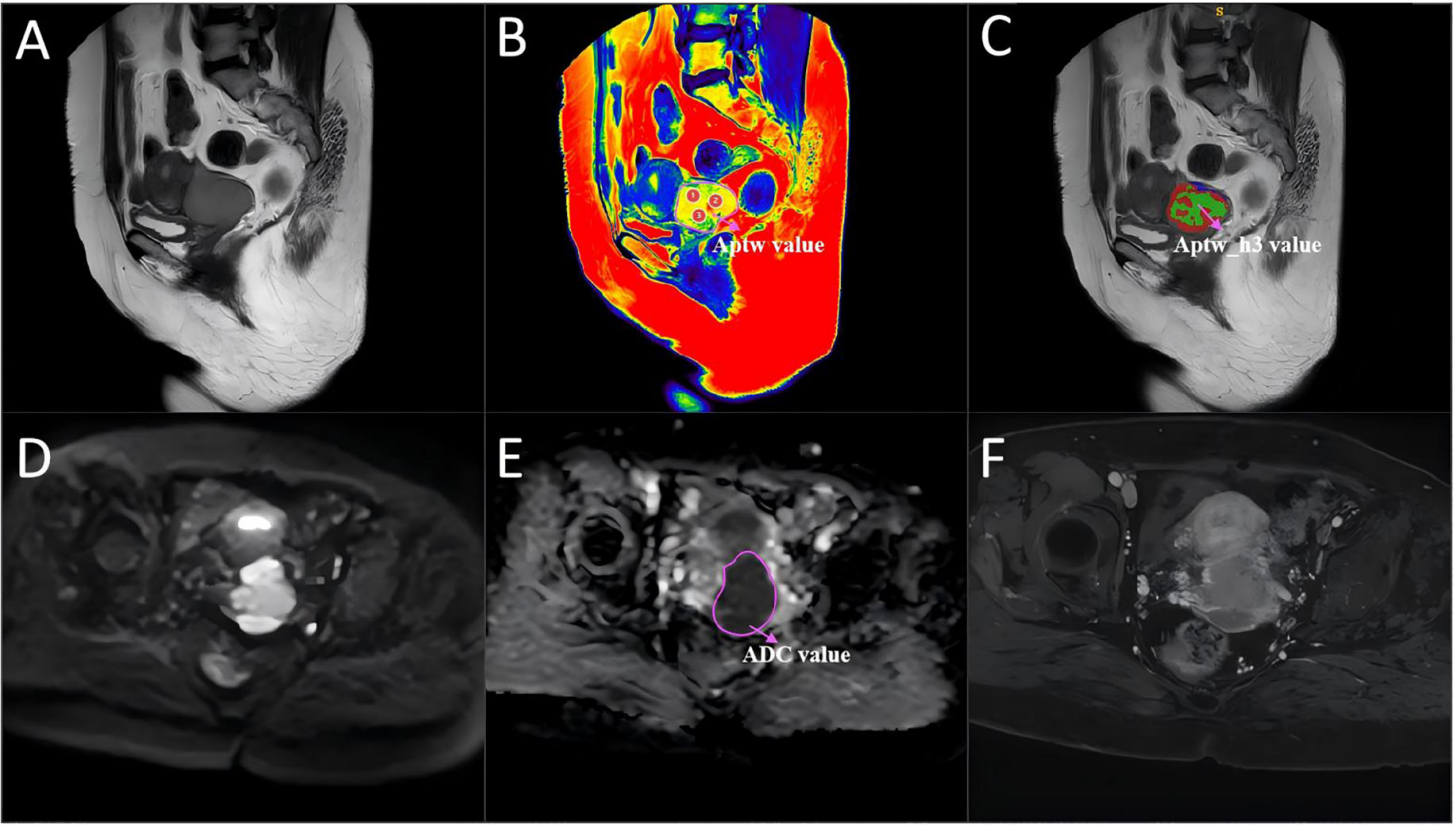
A 54-year-old woman with cervical cancer and LVSI, a serum CA125 level of 44.5 U/mL and a largest tumor diameter of 46 mm. **(A)** Sagittal T2WI. **(B)** APTw and T2W image fusion. **(C)** tumor region divided into three subregions with unsupervised clustering; red: subregion 1; blue: subregion 2; and green: subregion 3. **(D)** Diffusion-weighted image (b = 800 s/mm^2^). **(E)** ADC image. **(F)** DCE-MR image. The mean APTw and ADC values measured by the two radiologists were 4.36% and 0.83×10^−3^ mm^2^/sec, respectively. APTw, amide proton transfer-weighted; LVSI, lymphovascular space invasion.

The T2WI and APTw image sequences of the patients were uploaded to the Philips post-processing workstation. Two radiologists, with 5 and 15 years of experience in gynecological MRI respectively, independently reviewed all images and delineated the ROIs, blinded to the clinical and pathological information. Following the widely adopted International Standard Operating Procedures for APTw research (16), three circular ROIs were placed within the solid tumor portion on the APTw image slice corresponding to the sagittal T2WI slice showing the maximum tumor diameter. ROI placement carefully avoided blood vessels, necrotic areas, and the tumor stalk. The mean value of the measurements from these three ROIs was used for subsequent analysis (**Figures 3**, **4**). Additionally, the two radiologists blindly assessed the morphological and signal characteristics of the T2W images for 124 patients. In cases of disagreement, a consensus was reached through discussion.

### Habitat generation

To characterize intratumoral spatial heterogeneity, 13 local radiomic features were extracted at each voxel within the segmented tumor volume using a 3 × 3 × 3 sliding window. All voxels were then grouped into functional subregions using the K-means clustering algorithm. The optimal number of subregions (clusters) was determined for each tumor by systematically evaluating solutions with K ranging from 3 to 10, using the Calinski–Harabasz index as a selection criterion. For all cases, the highest index was consistently observed at K=3, justifying the use of three subregions. This approach resulted in three subregions, which were consistently color-coded as red (subregion 1), blue (subregion 2), and green (subregion 3) for visualization and subsequent analysis (**Figures 3**, **4**). Further technical details are provided in the **Supplementary Materials 1**.

### Feature extraction

In this study, we extracted handcrafted radiomic features from medical images, categorized into three types: (I) geometric shape, (II) first-order intensity, and (III) texture features. For each region of interest, 14 geometric shape features were first extracted. Following this, 20 different image transformation methods—including wavelet and Laplacian of Gaussian—were applied to the images. Each transformation produced 18 first-order intensity features and 75 texture features for each region.

### Feature selection

**Figure 5:**
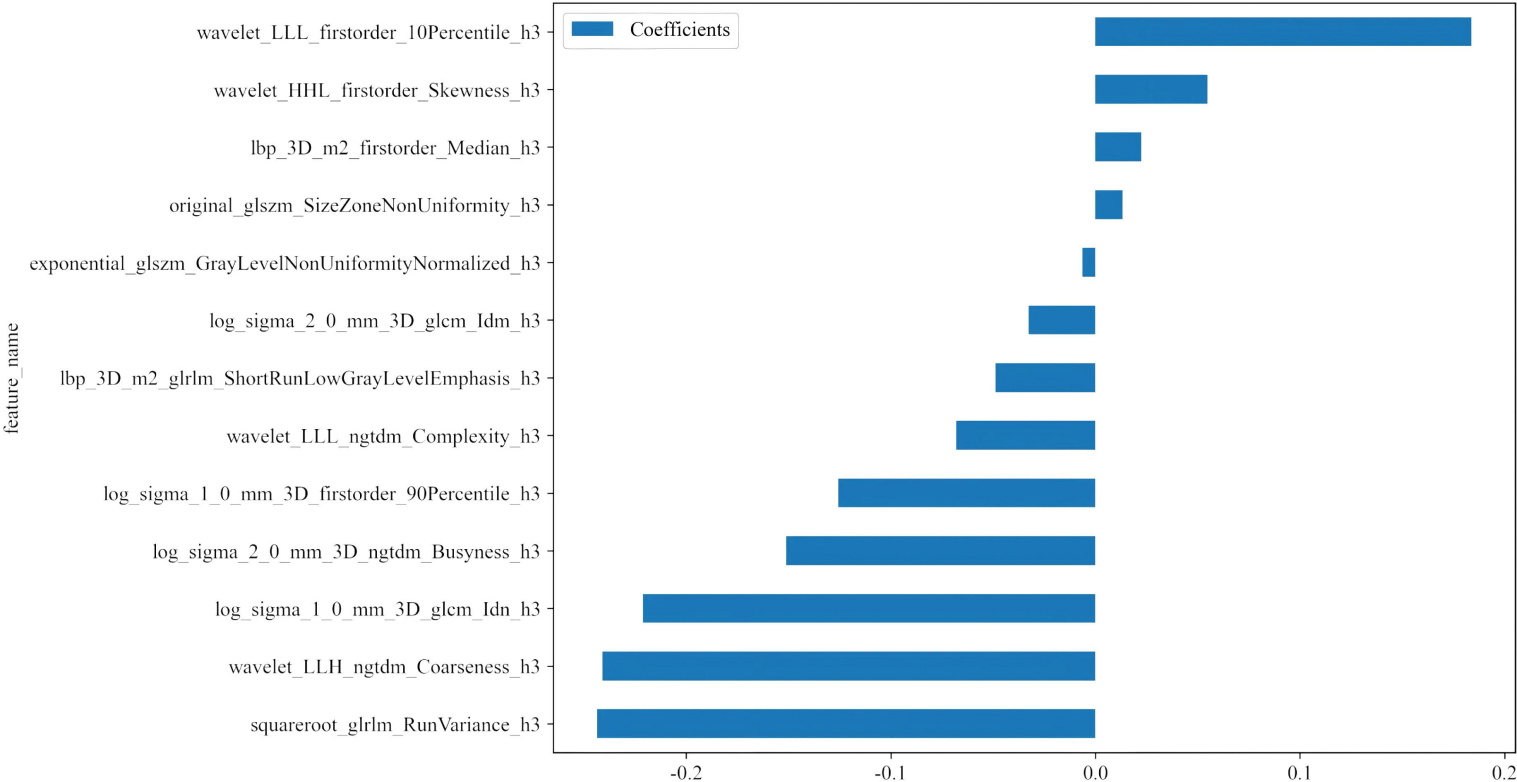
Histogram of the coefficients of the selected features. Thirteen optimal features were selected from the APTw_habitat 3 region. APTw, amide proton transfer-weighted.

To mitigate the impact of segmentation uncertainty, intra- and inter-rater reliability analyses were performed, and only features with an intraclass correlation coefficient (ICC) ≥ 0.85 were retained. To normalize the feature distribution, Z scores were calculated. Features significantly differentiating between groups (t test, *P* < 0.05) were retained to ensure relevance. To eliminate redundant features, Pearson correlation coefficients were computed, and a greedy recursive feature elimination strategy was applied. Further dimensionality reduction was performed using minimum redundancy maximum relevance (mRMR) followed by least absolute shrinkage and selection operator (LASSO) regression with tenfold cross-validation to determine the optimal λ value and select the most predictive features. The resulting features were linearly combined to calculate the radiomic score (APTw_h3) for each patient (**Figure 5**). After the final selection of radiomic features, we further compared the mean values of each selected feature between the LVSI-positive and LVSI-negative groups using the independent samples t-test.

### Observation indicators

Using the pathological examination results of all 124 CC patients as the gold standard, the sensitivity, specificity, and accuracy of the APTw features, APTw_h3, and the ADC in diagnosing CC LVSI were calculated and statistically compared. Receiver operating characteristic (ROC) curve analysis was conducted to evaluate the qualitative ability of the APTw features, APTw_h3, and the ADC to identify LVSI in CC.

### Model development and validation

To predict LVSI, three logistic regression models were established: a clinical–radiological model (based on CA125 and tumor size), a habitat radiomics model (based on selected APTw_h3 features), and a combined model incorporating all above features. Model development, including feature selection, normalization, training, and hyperparameter tuning, strictly followed a stratified 5-fold cross-validation framework, with all preprocessing performed exclusively on training folds to prevent information leakage. Hyperparameter optimization was implemented using nested cross-validation. Model evaluation relied on mean AUC, accuracy, and F1-score across validation folds. Full details are presented in the **Supplementary Materials**.

### Statistical analysis

All statistical analyses were performed using SPSS 25.0 (IBM) and MedCalc 23.1.1. The interobserver agreement for APTw and ADC measurements was assessed using the intraclass correlation coefficient (ICC), with ICC > 0.85 considered good. The Bland–Altman method was used to confirm measurement reproducibility. Categorical variables were expressed as frequency and percentage, and compared using the χ² test; continuous variables were compared by the Mann–Whitney U test. Diagnostic performance of imaging parameters and final models was further evaluated via ROC curve analysis, with sensitivity and specificity determined by the maximum Youden index. Comparisons of AUCs were performed using the DeLong test. A two-tailed *p* value < 0.05 was considered statistically significant.

## Results

### Participants

Among the 143 individuals who underwent imaging examinations, 8 participants excluded because their tumors had diameters of less than 1 centimeter or were invisible on MRI; 5 participants were excluded because of poor image quality and the presence of significant artefacts; 6 participants were excluded because of incomplete pathological information and clinical data; and 4 participants were excluded because they had received other treatments or interventions prior to surgery. A total of 124 participants with CC were included, with about 80% (99 patients) used for training and 20% (25 patients) for validation in each split (**Table 2**).

**Table 2:** Characteristics between the LVSI and Non-LVSI groups.

Variables	Total (N=124)	Non-LVSI group (N=74)	LVSI group (N=50)	*p*
Body mass index*	25.00 (22.61, 26.67)	24.96 (22.37, 26.53)	25.27 (23.29, 27.83)	0.261^†^
Age (years)*	50.00 (41.00, 55.00)	47.00 (41.00, 55.00)	51.00 (42.00, 58.00)	0.178^†^
Tumor size (mm)*	33.00 (31.00, 45.75)	31.00 (30.25, 36.25)	42.50 (31.00, 54.25)	<0.001^†^
CA125 (U/mL)*	22.50 (16.55, 44.50)	22.50 (14.80, 23.65)	44.45 (17.48, 48.33)	0.009^†^
FIGO stage				0.304^#^
≤II	112 (90.3%)	69 (93.2%)	43 (86.0%)	
>II	12 (9.7%)	5 (6.8%)	7 (14.0%)	
Menopausal status				0.268^#^
No	67 (54.0%)	43 (58.1%)	24 (48.0%)	
Yes	57 (46.0%)	31 (41.9%)	26 (52.0%)	
Myometrial Invasion				0.428^#^
<1/2	108 (87.1%)	63 (85.1%)	45 (90.0%)	
≥1/2	16 (12.9%)	11 (14.9%)	5 (10.0%)	
Pathological grade				0.273^#^
Low (1 or 2)	109 (87.9%)	67 (90.5%)	42 (84.0%)	
High (3)	15 (12.1%)	7 (9.5%)	8 (16.0%)	
Parametrial invasion				0.160^#^
No	88 (71.0%)	56 (75.7%)	32 (64.0%)	
Yes	36 (29.0%)	18 (24.3%)	18 (36.0%)	

CA125, cancer antigen 125; FIGO, International Federation of Gynecology and Obstetrics; Body mass index was calculated as participant weight in kilograms divided by participant height in meters squared; *Data are medians, with IQRs in parentheses; ^†^Mann-Whitney U test; ^#^χ^2^ test.

The interval between the MRI examinations and treatment ranged from 5 to 23 days. All participants underwent surgical treatment. Among the 124 participants, 74 (60%) were LVSI negative, whereas 50 (40%) were LVSI positive. The APTw_h3 value was significantly greater in the LVSI-positive group than in the LVSI-negative group (median of 0.33 [IQR, 0.28–0.41] vs. 0.67 [IQR, 0.44–0.78]; *P* < 0.001). Additionally, the levels of CA125 and the size of the tumor were both greater in the LVSI-positive group than in the LVSI-negative group (median values of 44.45 U/mL [IQR, 17.48–48.33 U/mL] and 31.00 mm [IQR, 30.25–36.25 mm], respectively, versus 22.50 U/mL [IQR, 14.80–23.65 U/mL] and 42.50 mm [IQR, 31.00–54.25 mm], respectively; both *P* < 0.001).

### Interobserver agreement

The intraclass correlation coefficients for the APTw features and ADC were 0.95 (95% CI: 0.92, 0.98) and 0.83 (95% CI: 0.80, 0.90), respectively. The Bland–Altman analysis indicated good reproducibility of the imaging parameters between the two radiologists (**Supplementary Figure S2**).

### Feature dimensionality and overfitting control

A total of 1,834 radiomic features were extracted from each tumor subregion (APTw_h1, APTw_h2, and APTw_h3), resulting in a combined total of 5,502 features. (**Supplementary Figure S3**). After a rigorous multi-step feature selection process (including intra- and interobserver ICC filtering, t-test, Pearson correlation analysis, mRMR, and LASSO regularization), only 13 optimal features, all from the APTw_h3 subregion, were retained for model construction. Features from APTw_h1 and APTw_h2 were completely excluded at the LASSO step, with all coefficients shrunk to zero. Therefore, the final predictive model utilized only 13 radiomic features in total (**Supplementary Table S1**). Pearson correlation heatmap of the selected features is shown in **Supplementary Figure S4**. Notably, the final feature-to-sample ratio (13 features for 124 patients) adheres to commonly accepted standards in radiomics modeling. Furthermore, the majority of the selected features demonstrated statistically significant differences between the LVSI-positive and LVSI-negative groups (all *P* < 0.05; **Supplementary Figure S5**), highlighting their strong discriminatory capacity.

### Comparison of the metrics for the APTw features, APTw_h3, and the ADC

**Figure 6:**
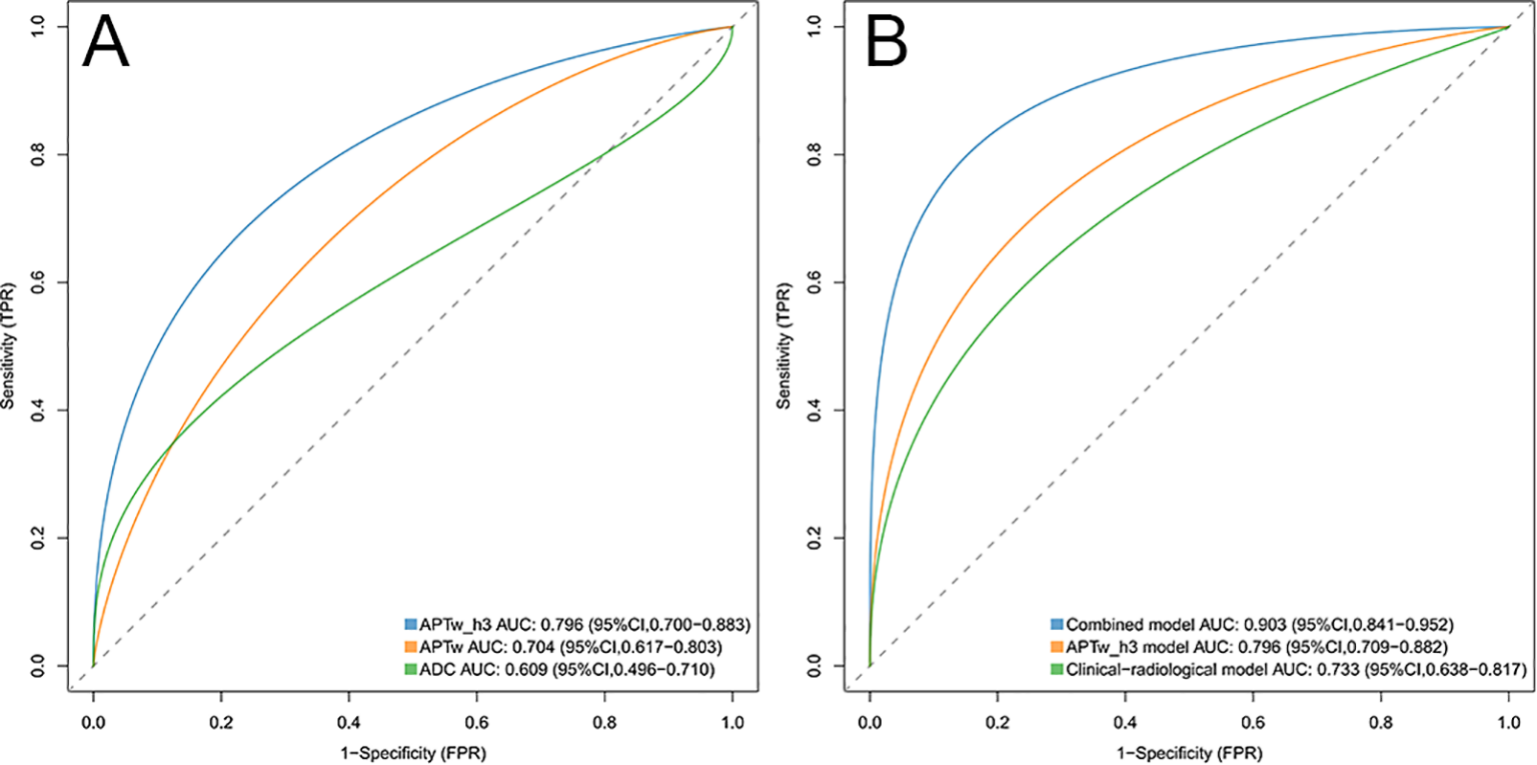
**(A)** ROC curves of the APTw_h3, APTw, and ADC values were used to predict lymphovascular space invasion positivity. The areas under the curve for these models were 0.796, 0.704, and 0.609, respectively. **(B)** ROC curves of the combined model, APTw_h3 model, and clinical-radiological model were used to predict positive lymphovascular space invasion. The areas under the curve for these models were 0.903, 0.796, and 0.733, respectively. ROC, Receiver operating characteristic.

**Figures 3** and **4** show the MRI scans of two CC patients without and with LVSI, respectively. The AUCs for the APTw features, APTw_h3, and the ADC in diagnosing LVSI in CC were 0.704, 0.796, and 0.609, respectively. The AUC for APTw_h3 was greater than that for the APTw and DWI features, as shown in **Figure 6A** and **Table 3**.

**Table 3:** Performance of imaging parameters in predicting lymphovascular space invasion.

Mertics	APTw	Aptw_h3	ADC
threshold	2.795	0.460	1.035
specificity	0.676	0.878	0.743
sensitivity	0.820	0.740	0.580
accuracy	0.734	0.823	0.677
npv	0.847	0.833	0.724
ppv	0.631	0.804	0.604
precision	0.631	0.804	0.604
recall	0.820	0.740	0.580
youden	0.496	0.618	0.323
z	4.607	6.768	2.276
*p*	<0.001	<0.001	0.023

npv, negative predictive value; ppv, positive predictive value.

### Risk factors for LVSI positivity

Univariable analysis revealed that the CA125 level (odds ratio [OR], 1.03 [95% CI: 1.01, 1.06]; *P* = 0.001), tumor size (OR, 1.05 [95% CI: 1.02, 1.09]; *P* = 0.001), and APTw_h3 values (OR, 7.99 [95% CI: 7.61, 9.78]; *P* < 0.001) were associated with LVSI positivity (**Table 4**). After variables with *P* < 0.05 in the univariable analysis were included in the multivariate analysis, the CA125 level (OR, 1.09 [95% CI: 1.04, 1.14]; *P* < 0.001), tumor size (OR, 1.06 [95% CI: 1.03, 1.10]; *P* < 0.001), and APTw_h3 values (OR, 9.40 [95% CI: 7.98, 12.26]; *P* < 0.001) were retained as independent predictors of LVSI positivity (**Table 4**).

**Table 4:** Univariable and multivariable logistic regression analyses in predicting lymphovascular space invasion.

Characteristics	Univariable Analysis	P Value	Multivariable Analysis	*P* Value
Age (y)	1.02(0.98,1.06)	0.17		
Body mass index	1.08(0.98,1.17)	0.08		
CA125 (U/mL)	1.03(1.01,1.06)	0.001	1.09(1.04,1.14)	<0.001
Tumor size (mm)	1.05(1.02,1.09)	0.001	1.06(1.03,1.10)	<0.001
APTw_h3	7.99(7.61,9.78)	<0.001	9.40(7.98,12.26)	<0.001

CA125, cancer antigen 125; APTw, amide proton transfer-weighted.

### Assessment of risk models

The clinical-radiological model predicted LVSI positivity with an AUC of 0.733 (95% CI: 0.638, 0.817), which is comparable to the AUC of the model that included only the APTw_h3 values (0.796 [95% CI: 0.709, 0.882]; cut-off value, 0.46; *P* = 0.39). The combined model for predicting LVSI positivity had an AUC of 0.903 (95% CI: 0.841, 0.952), which was greater than that of the clinical-radiological model (*P* < 0.001) and the APTw_h3 model (*P* < 0.001). Furthermore, the addition of APTw_h3 data to the clinical-radiological model resulted in higher sensitivity (88% vs. 82%) and specificity (83.8% vs. 64.9%) in determining LVSI positivity than did the use of the clinical-radiological model alone (**Tables 5**, **6**, **Figure 6B**). Detailed performance metrics obtained from 5-fold cross-validation are shown in **Supplementary Table S2**.

**Table 5:** Comparison of the model AUCs in predicting lymphovascular space invasion.

Comparisons	AUC1	AUC2	Diff	z	*p*
Combined model vs Aptw_h3 model	0.90 (0.84,0.95)	0.80 (0.71,0.88)	0.09 (0.02,0.16)	2.64	<0.001
Combined model vs Clinical-radiological model	0.90 (0.84,0.95)	0.73 (0.64,0.82)	0.16 (0.06,0.25)	3.40	<0.001
Aptw_h3 model vs Clinical-radiological model	0.81 (0.72,0.90)	0.73 (0.64,0.82)	0.06 (-0.08,0.20)	0.85	0.39

CA125, cancer antigen 125; APTw, amide proton transfer-weighted.

Clinical-radiologic model was based on CA125 and tumor size.

Combined model was based on CA125, tumor size, and APTw_h3.

**Table 6:** Comparison of model metrics in predicting lymphovascular space invasion.

Mertics	Combined model	Aptw_h3 model	Clinical-radiological model
threshold	0.357	0.460	0.325
specificity	0.838	0.878	0.649
sensitivity	0.880	0.740	0.820
accuracy	0.855	0.823	0.718
npv	0.912	0.833	0.842
ppv	0.786	0.804	0.612
precision	0.786	0.804	0.612
recall	0.880	0.740	0.820
youden	0.718	0.618	0.469
z	14.964	6.768	5.344
*p*	<0.001	<0.001	<0.001

npv, negative predictive value; ppv, positive predictive value; CA125, cancer antigen 125; APTw, amide proton transfer-weighted.

Clinical-radiologic model was based on CA125 and tumor size.

Combined model was based on CA125, tumor size, and APTw_h3.

## Discussion

This study innovatively applied habitat radiomics analysis based on APTw MRI to predict LVSI in patients with CC, achieving valuable outcomes. To better demonstrate the superiority of this novel approach, we compared it with several existing methods commonly used in the field, solidifying its position as a more effective and reliable prediction tool.

Previous research (20–23) has indicated that tumors with LVSI generally display stronger invasiveness, a greater number of microvessels, and higher cellular metabolic activity, which can cause a substantial increase in APT signals. Nevertheless, conventional APTw signal measurement only centers on the maximum cross - section of the tumor, capturing information from a single plane and neglecting the internal heterogeneity of the tumor. Moreover, the widely - used ADC analysis method also has its drawbacks. The study by Cheng et al. (24) on the correlation between LVSI and ADC values differs from our findings. We believe this inconsistency may be attributed to the complex and uncertain impact of tumor stromal components on ADC values. Some tumors generate more fibrotic stroma (25), which may affect osmotic pressure, restrict water diffusion, and result in reduced ADC values, thereby masking the differences in LVSI status.

In contrast, habitat analysis divides the tumor into diverse sub - regions, enabling a more precise reflection of the biological characteristics of different areas within the tumor. In this study, we discovered that the features extracted from sub - region 3 (APTw_h3), which exhibits relatively high signal intensity on APTw imaging, have excellent predictive performance for LVSI. In contrast, the features from sub - regions 1 and 2 show no significant differences between the LVSI and non - LVSI groups. This might be because sub - region 3 is enriched with tumor cell sub - populations possessing higher invasive and metastatic potential. These cell sub - populations may exhibit special biological behaviors, such as enhanced motility and elevated angiogenesis ability, making it easier for them to penetrate the lymphovascular space and trigger LVSI. The biological link between tumor heterogeneity and LVSI is well - established. Tumor cells with distinct genetic and phenotypic profiles within different sub - -regions lead to varying levels of invasiveness. The high - risk sub - populations in APTw_h3 are more likely to break through the barriers and enter the lymphovascular system, which is why our habitat radiomics analysis can effectively predict LVSI by capturing these heterogeneities. In contrast, simple APTw imaging and ADC analysis find it difficult to capture these internal heterogeneous differences within the tumor, thus failing to accurately predict LVSI.

Taking the features we selected as an illustration, such as wavelet_LLL_firstorder_10Percentile_h3 and wavelet_HHL_firstorder Skewness_h3, these features may mirror certain biological characteristics of tumor cells. Features with positive coefficients may be associated with biological processes that promote tumor cell invasion and metastasis. For example, a high 10th percentile may imply the existence of a subset of cells with high metabolic activity or proliferation ability within the tumor, which are more prone to breaking through the basement membrane and entering the lymphovascular space. Features with negative coefficients, like log_sigma_2_0_mm_3D_ngtdm_Busyness_h3, may be related to factors that inhibit tumor invasion and metastasis. For instance, this feature may reflect certain components in the tumor microenvironment that suppress cell motility or angiogenesis. These detailed feature information are challenging to be reflected in simple APTw imaging and ADC analysis, and habitat radiomics analysis can uncover this valuable information, offering a more comprehensive foundation for LVSI prediction.

Through a rigorous feature selection method, this study ultimately determined the APTw_h3 - related features for constructing the prediction model. Multivariate analysis demonstrated that tumor size, CA125 level, and APTw_h3 value were significantly correlated with LVSI positivity (odds ratio, 1.09 - 9.40; all P < 0.001). The combined model (AUC = 0.903) performed significantly better than the clinical - radiological model (AUC = 0.733) and the APTw_h3 model (AUC = 0.796) in predicting LVSI. This indicates that integrating APTw_h3 - related features into the clinical - radiological model can significantly enhance the prediction accuracy of LVSI. Biologically, tumor size reflects the growth extent of the tumor. Larger tumors may harbor more invasive cell sub - populations, thereby increasing the risk of LVSI. CA125 is a tumor marker, and an elevated level may be linked to tumor cell proliferation, invasion, and metastasis. The habitat features represented by APTw_h3 more comprehensively reflect the heterogeneity and biological characteristics of the tumor, capturing information that traditional indicators cannot, and thus can provide more accurate LVSI prediction when combined with tumor size and CA125 level.

The analysis of clinicopathological features revealed that the tumor size and CA125 level in the LVSI - negative group were significantly lower than those in the LVSI - positive group (both P = 0.001). These results are consistent with those of previous reports (26, 27). A previous study by Chen et al. (28) compared tumor sizes between LVSI - positive and LVSI - negative groups in 315 women with CC and reported that the maximum tumor diameter in LVSI - negative CC was lower than that in LVSI - positive CC. Recently, Xu et al. (29) analyzed the data of 40 CC patients (72.5% LVSI - positive and 27.50% LVSI - negative). Their results revealed that CA125 levels in the LVSI - negative group were significantly lower than those in the LVSI - positive group and that elevated preoperative CA125 levels were associated with an increased risk of LVSI positivity.

Currently, the diagnosis of LVSI mainly relies on postoperative histopathological analysis, which is invasive and has certain limitations. The prediction model based on APTw habitat radiomics analysis proposed in this study can offer doctors a more accurate preoperative risk assessment of LVSI, facilitating the formulation of personalized treatment plans. For example, for patients predicted to be LVSI - positive, more aggressive treatment strategies, such as extended surgical resection and adjuvant chemotherapy, can be adopted; for patients predicted to be LVSI - negative, overtreatment can be avoided, reducing patients’ suffering and the waste of medical resources. In contrast, simple APTw imaging and ADC analysis have a weaker ability in preoperative LVSI prediction and cannot provide such precise information for clinical decision - making.

Although this study yielded encouraging results, several limitations should be acknowledged. First, the current workflow involves multiple manual preprocessing steps and ROI segmentation, which limits automation and poses challenges for large-scale clinical application. Second, the extraction of radiomic features is sensitive to imaging parameters and acquisition protocols, underscoring the need for standardizing and optimizing the analysis pipeline to improve adaptability across different equipment and conditions. In addition, as this study was conducted at a single center with a limited and relatively homogeneous sample, the generalizability of the model has yet to be fully validated. Therefore, our findings should be considered exploratory, and further validation in larger, independent external cohorts is required to establish the robustness and clinical value of the models. Furthermore, practical deployment of machine learning models must take into account risks such as data bias, overfitting, and relevant ethical or legal challenges. Finally, while the proposed non-invasive method shows promise as an adjunct, it cannot replace biopsy, which remains the gold standard for diagnostic accuracy.

In conclusion, this study demonstrates that the habitat radiomics feature (APTw_h3) derived from APTw imaging serves as an independent predictor of LVSI positivity in cervical cancer patients. By integrating habitat radiomics features with conventional clinicoradiological factors, our model achieved improved predictive accuracy for LVSI, providing significant support for preoperative risk stratification and individualized clinical decision-making. These results highlight the potential value of habitat-based radiomics analysis in enhancing non-invasive tumor characterization. Future work will focus on further automating the workflow, establishing standardized imaging and feature extraction protocols, and performing external validation in large, diverse cohorts to facilitate clinical translation and wide adoption of this approach.

## Data Availability

The original contributions presented in the study are included in the article/**Supplementary Material**. Further inquiries can be directed to the corresponding author.
