# Supplementary material for: Amide proton transfer-weighted habitat radiomics: a superior approach for preoperative prediction of lymphovascular space invasion in cervical cancer
Source: Front Oncol. 2025 Jul 10;15:1599522. doi: 10.3389/fonc.2025.1599522 (PMC12286790; doi:10.3389/fonc.2025.1599522)
Supplement: Supplementary file 1 [file DataSheet1.docx]

**Supplementary Materials**

**Supplementary Materials 1: Habitat Generation and Feature extraction**

We extracted local features from each voxel using a 3×3×3 moving window to capture intensity, texture, and statistical properties. Thirteen radiomic features were computed per voxel:

- firstorder_MeanAbsoluteDeviation (MAD): Represents the average distance of all intensity values from the image’s mean value, defined as MAD =
- Difference Entropy: Assesses the variability and heterogeneity in neighbourhood intensity values and is defined as difference_entropy =

along with firstorder_Median, original_glcm_JointEnergy, original_glcm_Imc1, original_glcm_DifferenceVariance, original_glcm_InverseVariance, original_glcm_JointEntropy, original_glrlm_LongRunEmphasis, original_glszm_SizeZoneNonUniformityNormalized, original_glszm_SmallAreaHighGrayLevelEmphasis, original_ngtdm_Strength, and original_ngtdm_Contrast (computation methods detailed in PyRadiomics documentation [[http://pyradiomics.readthedocs.io](http://pyradiomics.readthedocs.io/)]).

For habitat identification, voxel-wise feature vectors were subjected to K-means clustering (scikit-learn v1.2.2), with systematic testing for cluster numbers (K=3 to 10). K=3 was chosen in all cases based on the maximum Calinski-Harabasz index, assigning each voxel to one of three subregions (subregion 1=red, subregion 2=blue, subregion 3=green). Clustering was run with n_init=20 and random_state=42 to ensure reproducibility. All processing was implemented in Python 3.1, combining PyRadiomics and scikit-learn, with custom code available upon request.

This specific feature set was selected based on the methodology established by Wu et al. [20], who demonstrated that this combination effectively captures local heterogeneity patterns relevant to tumor biology and microenvironment. Given our similar objectives in characterizing intratumoral heterogeneity, we adopted this validated feature set for habitat analysis.

- Wu J, Meng H, Zhou L et al (2024) Habitat radiomics and deep learning fusion nomogram to predict EGFR mutation status in stage I non-small cell lung cancer: a multicenter study. Sci Rep 14:15877

| 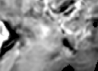  APTw image | 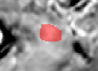  APTw mask | 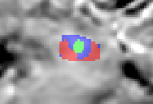  APTw subregions | 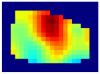  original_firstorder_MeanAbsoluteDeviation |
| --- | --- | --- | --- |
| 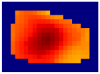  original_firstorder_Median | 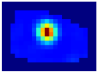  original_glcm_JointEnergy | 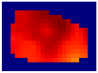  original_glcm_DifferenceEntropy | 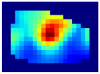  original_glcm_DifferenceVariance |
| 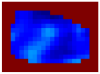  original_glcm_Imc1 | 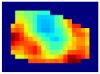  original_glcm_InverseVariance | 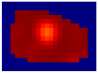  original_glcm_JointEntropy | 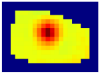  original_glrlm_LongRunEmphasis |
| 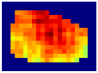  original_glszm_SizeZoneNonUniformityNormalized | 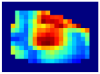  original_glszm_SmallAreaHighGrayLevelEmphasis | 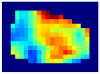  original_ngtdm_Strength | 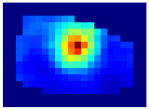  original_ngtdm_Contrast |

**Fig. S1.** Generated habitat regions and their characteristics.

| 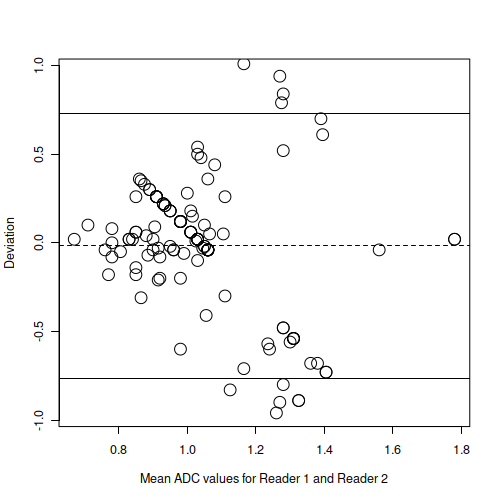  A | 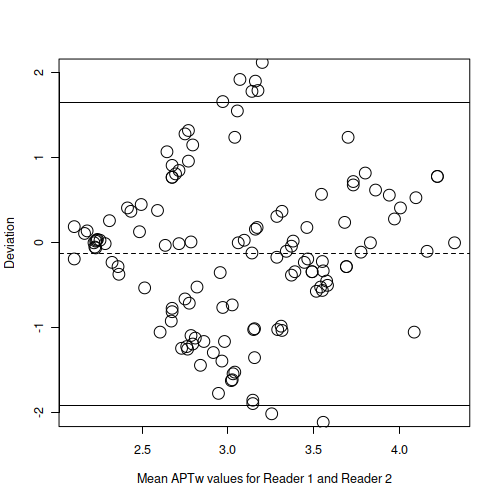B |
| --- | --- |

**Fig. S2.** Bland‒Altman plots comparing the reproducibility of the ADC and APTw measurements from the two independent readers. (A) Plot showing that for the ADC (mm^2^/s), the mean difference was -0.01 mm^2^/s (95% CI: −0.75, 0.75). (B) Plot showing that for the APTw value, the mean difference was -0.02 (95% CI: −1.98, 1.68). The dotted line represents the mean difference, and the upper and lower solid lines represent the upper and lower limits of agreement, respectively.


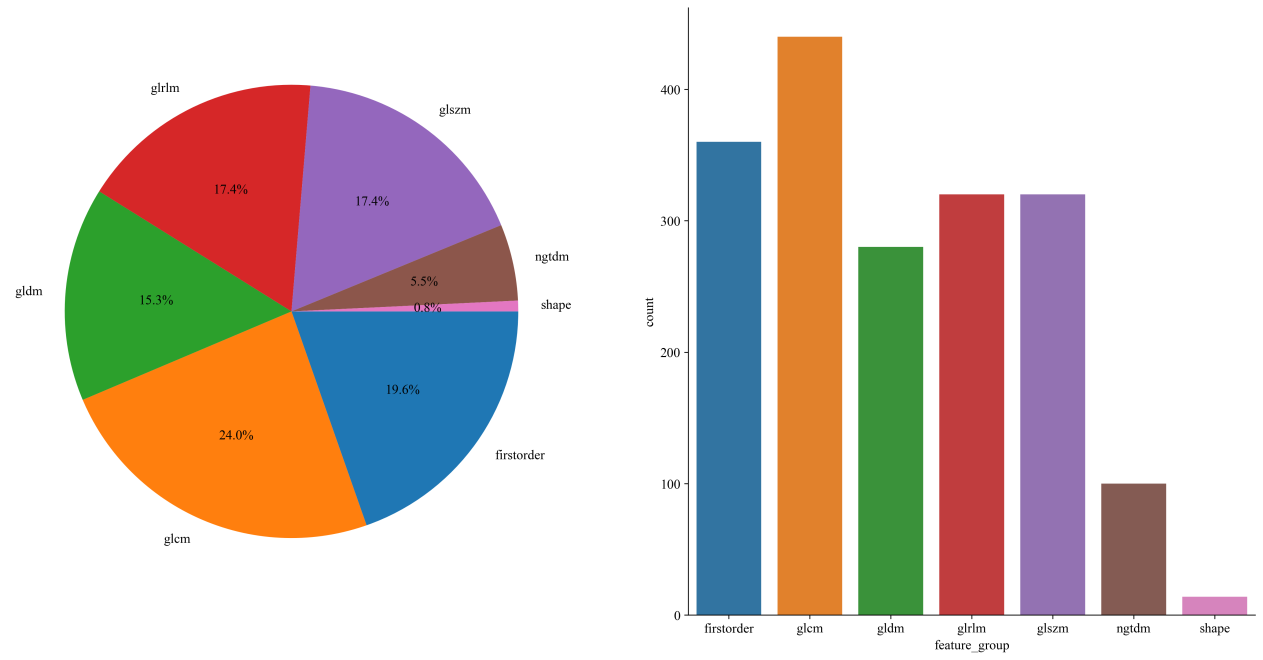


**Fig. S3.** Generated handcrafted features (ratios and counts).


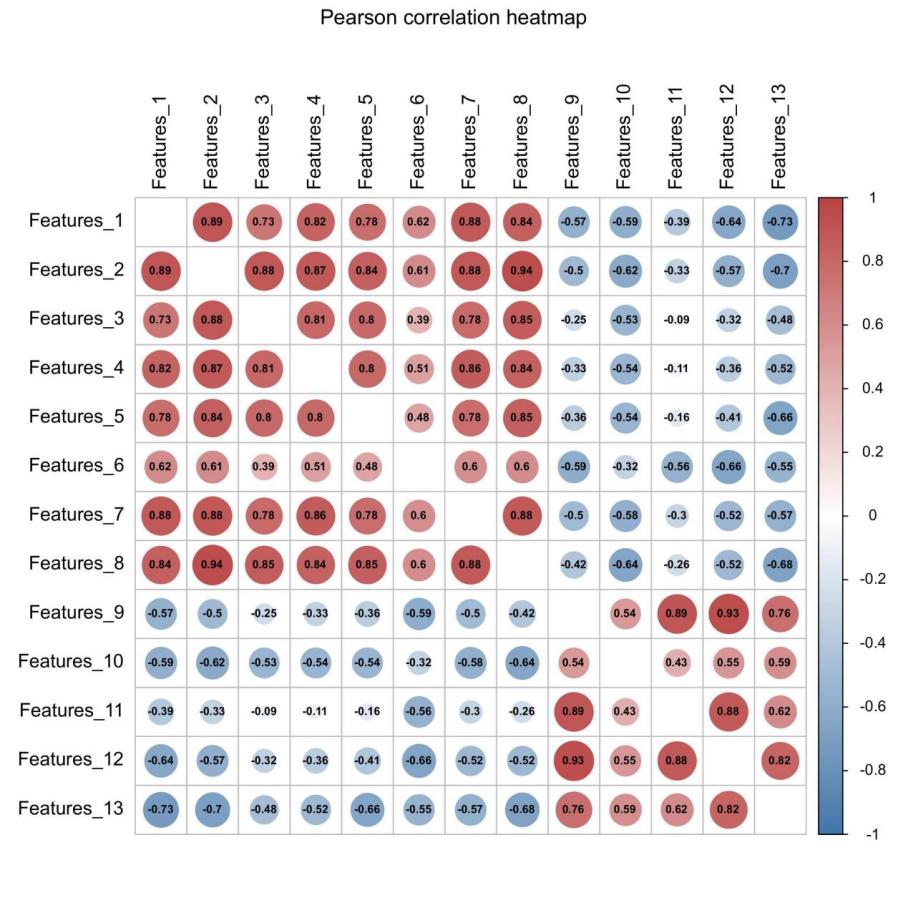


**Fig. S4.** Heatmap of Pearson correlation coefficients among selected features.


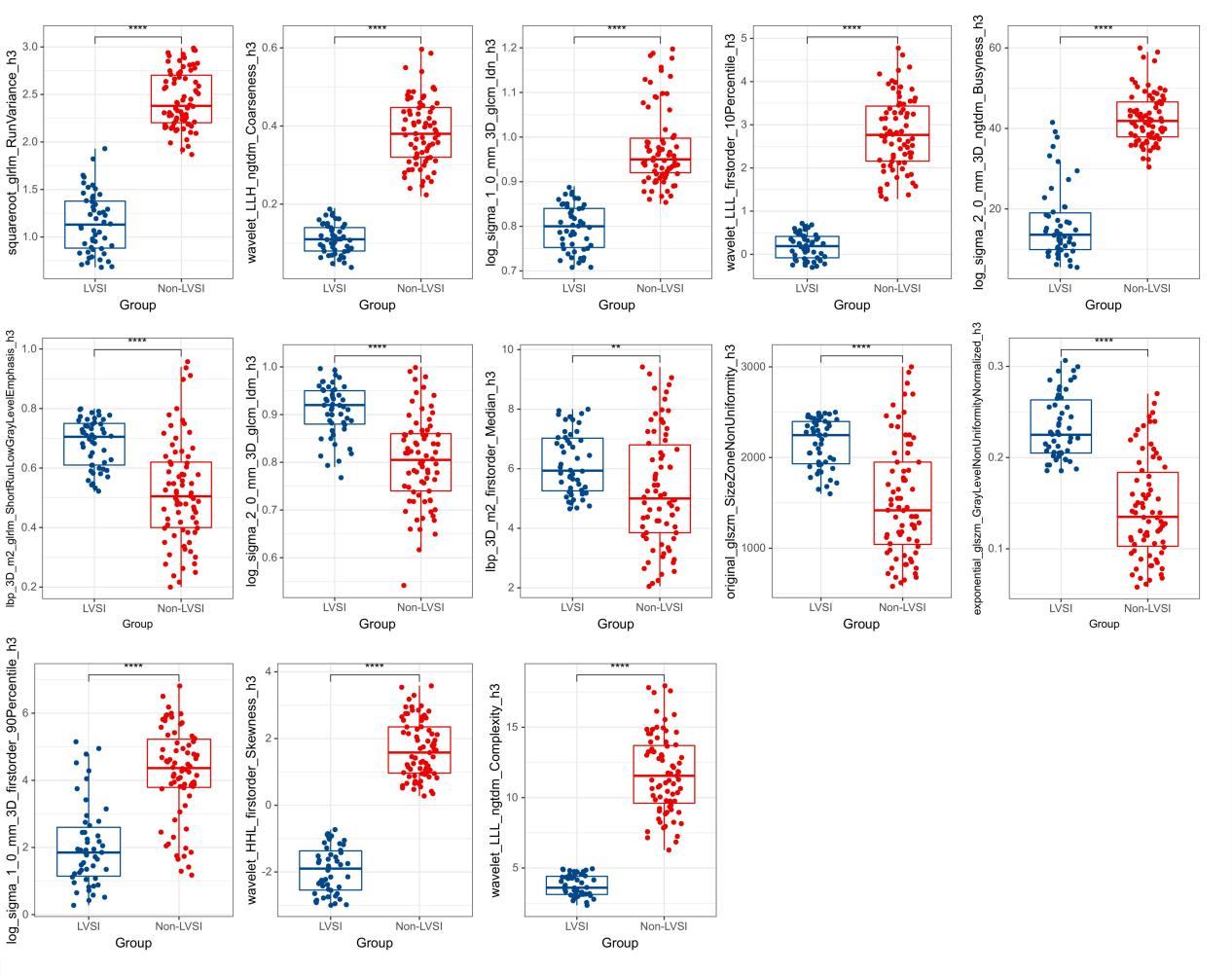


**Fig. S5.** Group-wise boxplots of the 13 selected APTw_h3 radiomic features between LVSI-positive and LVSI-negative groups (t-test).

**Table S1.** Progressive Feature Selection Workflow and Remaining Feature Counts

| Selection Step | APTw_h1 | APTw_h2 | APTw_h3 |
| --- | --- | --- | --- |
| Initial Extraction | 1834 | 1834 | 1834 |
| Post-ICC Filtering | 1210 | 1295 | 1257 |
| Post-t-test | 305 | 312 | 320 |
| Post-RFE/Correlation | 59 | 62 | 60 |
| Post-mRMR | 19 | 18 | 20 |
| Post-LASSO | 0 | 0 | 13 |

**Supplementary Materials 2: Feature Selection Methods and Rationale**

A rigorous, multi-stage feature selection strategy was employed to optimize model performance while ensuring interpretability and generalizability. The rationale and contribution of each method are as follows:

1. **Intra- and Inter-rater Reliability (ICC, cutoff 0.85):**

To ensure robustness to segmentation variability, features were retained only if their intraclass correlation coefficient (ICC) across raters exceeded 0.85. This step excluded features sensitive to manual delineation, improving reproducibility.

1. **Univariate Relevance Filtering (t test, *P* < 0.05):**
   To enhance the discriminatory power of the radiomic signature, features demonstrating statistically significant differences between groups were retained based on Student’s t test. This ensured that selected features captured group-related variance likely relevant to the outcome.
2. **Redundancy Reduction (Pearson Correlation + Recursive Elimination):**
   Highly correlated features were identified via Pearson correlation coefficients (threshold: r > 0.9), since redundant features may introduce noise and multicollinearity. A greedy recursive feature elimination approach further ensured the selection of uncorrelated features, simplifying model interpretation and mitigating overfitting.
3. **Multivariate Selection (mRMR and LASSO):**
   Minimum redundancy maximum relevance (mRMR) ranked features to balance high relevance to the outcome and low mutual redundancy. Subsequently, least absolute shrinkage and selection operator (LASSO) regression with tenfold cross-validation performed further dimensionality reduction by penalizing less informative features, ultimately identifying a parsimonious set of nonzero-coefficient predictors for modeling.

**Supplementary Materials 3: Model development pipeline**

For each fold in the stratified 5-fold cross-validation, approximately 80% of the data were allocated to training and 20% to validation. Feature normalization (z-score standardization) and selection (based on t-tests, Pearson correlation, mRMR, and LASSO regularization) were performed strictly within the training data. Parameters derived from the training fold were then applied to the validation fold only. Hyperparameters were optimized via a nested 3-fold cross-validation within each training fold. At no step was the validation fold used during feature or parameter selection, ensuring no data leakage. Logistic regression implementation used Scikit-learn (v.1.2.2) and all performance metrics represent mean values over validation folds.

**Table S2.** Model performance across 5-fold cross-validation

| **Model** | **AUC (mean±SD)** | **Accuracy (mean±SD)** | **F1-score (mean±SD)** |
| --- | --- | --- | --- |
| Clinical-radiological model | 0.733±0.06 | 0.718±0.04 | 0.71±0.05 |
| APTw_h3 model | 0.796±0.09 | 0.823±0.05 | 0.80±0.04 |
| Combined model | 0.903±0.04 | 0.855±0.03 | 0.85±0.03 |

Note: All values are reported as mean (± standard deviation) across five stratified cross-validation folds.
